# An Approach to Improving Compliance of Treatment in Asymptomatic Bacteriuria

**DOI:** 10.51894/001c.38898

**Published:** 2023-12-05

**Authors:** Johnathan Lewis, Angelic Dye, Tracy Koehler, Justin Grill, Sarah Baribeau, Caleb Bryant

**Affiliations:** 1 Graduate Medical Education, Emergency Medicine Residency Program Mercy Health Muskegon; 2 Scholastic Activity Support Mercy Health Muskegon; 3 Administration Mercy Health Muskegon; 4 Medical Student MSU College of Osteopathic Medicine; 5 Pharmacy Mercy Health Muskegon

**Keywords:** ASB, UTI, compliance, spaced repetition, antibiotics

## Abstract

**INTRODUCTION:**

Asymptomatic bacteriuria (ASB) is the presence of bacteria in the urine without attributable signs or symptoms of a urinary tract infection (UTI). This condition is often inappropriately treated per the 2019 Infectious Disease Society of America guidelines. This quality improvement project aimed to reduce improper treatment of ASB via a three-phase spaced repetition approach over a 12-month 2021-2022 period within a Michigan emergency department (ED), targeting 43 ED clinicians.

**METHODS:**

During Phase I, a 20-minute teleconference educational intervention was delivered by an Infectious Disease physician and pharmacist. During Phase II, a “hard stop” was implemented within the electronic health record preventing reflex urinalysis culture without indication. During Phase III, a latent period of no intervention took place. The authors’ goal was to achieve > 80% compliance to ASB treatment guidelines.

**RESULTS:**

Overall compliance after the project initiative was 66.7%, an absolute increase of 16.7% from baseline compliance. Using data from 54 patients, this represented a statistically significant (p = 0.01) increase from baseline but fell short of the target of > 80%.

**DISCUSSION:**

Although the authors fell short of their goal of a 30% increase, data from the project suggests a spaced repetition approach to education and workflow changes could be an effective method to increasing medical provider compliance with treatment of ASB.

**CONCLUSION:**

Identifying the ideal strategy to change treatment patterns of ED clinicians for ASB to align with guidelines remains key. There is still a need for ongoing efforts in this realm for progress to be made. Keywords: asymptomatic bacteriuria, urinary tract infection, compliance, spaced repetition, antibiotics.

## INTRODUCTION

Asymptomatic bacteriuria (ASB) is the presence of bacteria in the urine without attributable signs or symptoms of a urinary tract infection (UTI).^1^ ASB has been widely identified as a clinical entity known to be inappropriately treated, especially in emergency department (ED) settings.^2–4^ Various outside factors including hospital culture or patient census may place undue stress on ED clinicians to identify etiologies of relatively unclear causes of clinical decline in patient populations, particularly in those who are elderly or demented.^5–8^ This may lead to early closure and inaccurate identification of abnormal urinalysis results, subsequently leading to unnecessary courses of antibiotics that ultimately negatively influence patient outcomes.

With the latest 2019 Infectious Disease Society of America (IDSA) guidelines referencing ASB, experts encouraged seeking alternative sources for clinical decline along with a strong recommendation against unnecessary antibiotics which have been known to cause complications themselves (e.g., antibiotic resistance, side effects, and *Clostridioides difficile* infection).^1^ Current efforts to combat non-compliance with treatment of ASB are underway at many hospitals throughout the country, and antibiotic stewardship interventions have been identified as being an effective means to encourage appropriate management.^9^

A local analysis of appropriate treatment of ASB completed over a period of 6 months at the authors’ community-based hospital ED, conducted in conjunction with clinical pharmacists, infectious disease specialists, and emergency clinicians, had revealed a suboptimal compliance rate of 50%. This finding, after publication of the 2019 IDSA ASB guidelines update, prompted the development and implementation of a quality initiative based educational intervention at the authors’ institution.

### Purpose of Project

The primary goal of the project was to increase ASB antibiotic ordering compliance to >80% post-intervention using a stepwise educational intervention approach.

## METHODS

This project was undertaken as a clinical quality improvement initiative at Mercy Health and, as such, was not formally supervised by the Mercy Health Regional Institutional Review Board per their policies. A stepwise educational intervention using a spaced repetition strategy was developed for the study. Spaced repetition in the world of education has long been identified as an effective means to reinforce concepts and influence long term retention.^10,11^

Using this approach, a basic framework as laid out by the Infectious Diseases Society of America (IDSA) in addressing ASB was used by the authors to provide ED clinicians with a framework of reference for appropriate treatment and antibiotic stewardship. The project also included changes to the way urine studies were ordered in the electronic health record (EHR). Three phases were implemented, with compliance assessment after each phase, and overall compliance assessment for the entirety of the project intervention.

### Phase I

The first step of the intervention was initiated with an educational teleconference delivered by an infectious disease physician and infectious disease pharmacist. This presentation was pre-recorded and distributed among a total of 43 providers practicing in the authors’ local ED. The presentation included pertinent definitions and several case studies identifying appropriate and inappropriate usage of antibiotic therapy by ED clinicians in the context of bacteriuria.

Copies of the original IDSA guideline paper on ASB were additionally distributed for individual reference. In conjunction, practical quick reference flow charts published by Loeb et al. were provided and posted throughout the emergency department to further solidify concepts in appropriate testing and treatment.^12^ These interventions took place over the period of one month. This was followed by a period of three months where no further education or prompts were made. Data from this timeframe was then compiled and later assessed.

### Phase II

After the completion of the first intervention period and its following latent period, the second intervention took place. Previously in the EHR, a urinalysis with reflex microscopic and urine culture could be ordered without specific justification. To change this process and avoid orders of unnecessary urine culture, a hard stop was put into place with a urinalysis with reflex culture order. This required clinicians to select why the order was indicated, as there were defined criteria that needed to be met for appropriate ordering in the context of a patient demonstrating signs or symptoms consistent with a UTI or potential urinary source for systemic infection. (Table 1).

**Table 1.** Justification of urine culture ordering

**a. Culture indicated by documentation of localized genitourinary symptoms (1 or more of the following):**
Urinary frequency
Urinary urgency
Dysuria
Costovertebral angle tenderness
Hematuria
Suprapubic pain or tenderness
**b. Culture indicated by documentation of non-localized genitourinary symptoms:**
Fever (≥38℃), rigors, or hypothermia without alternate explanation
New onset mental status change with potential signs of infection (WBC >10 x 10⁹), or hypotension (systolic blood pressure <90 mmHg), or >2 SIRS criteria
Renal transplant <1 month prior to obtained culture
Pregnancy without signs or symptoms of UTI
Pre-procedure screening for upcoming urologic procedure with anticipated urologic mucosal trauma

Without selecting an indication, the order for urine culture could not be placed. The defined timeline of intervention during this period was one month as ED clinicians developed familiarity with the process. Clinicians were also informed of the EHR change upon its implementation along with justification of a new process. After a one-month period, a latent period of three months (i.e., during which no intervention occurred) took place in similar fashion. Data were compiled from this period and later assessed by two of the investigators (JL and AD).

### Phase III

The third phase of the intervention involved a period without any educational prompts or changes. This was again followed by another three-month latent period, consistent with timelines of the previous two interventions. Data from this period were compiled and assessed in a similar fashion to earlier project phases.

### Study Population

Patient cases were included for analyses if they had a positive urine culture and were ≥18 years of age. A total of 174 cases were initially identified. Upon review, 120 (68.9%) patients with UTI symptoms were excluded as they did not meet empiric criteria for ASB. The remaining 54 (31.0%) cases were then determined to be eligible based on chart review when evaluated against the pre-specified criteria (Table 1b).

### Compliance Assessment

Compliance was defined as appropriate treatment of patients in meeting the definition of ASB and appropriately receiving antibiotics for the previously described indications (Table 1b) or cases meeting the definition of ASB and not receiving antibiotics if none of these indications were met, based off the IDSA guidelines.^1^ Cases of ASB that did not receive appropriate management were determined to be non-compliant.

### Analyses

Summary statistics were calculated. Quantitative data are reported as mean + SD and nominal data are reported as percentages. Percent increase/decrease were calculated for overall compliance compared to baseline, as well as for compliance for each phase of the intervention. The binomial test was used to assess for statistical differences in percent compliance between baseline and post-intervention. Chi-square or Fisher’s exact test were used to assess compliance rates between intervention phases. Significance was assessed at p<0.05. Data were analyzed by author TJK using IBM SPSS Statistics, v 25 (Armonk, NY: IBM Corp).

## RESULTS

The average age of the study sample (n=54) was 71.2±18.2 years with 32 (59.3%) reporting female sex.

### Compliance

**Figure 1.**
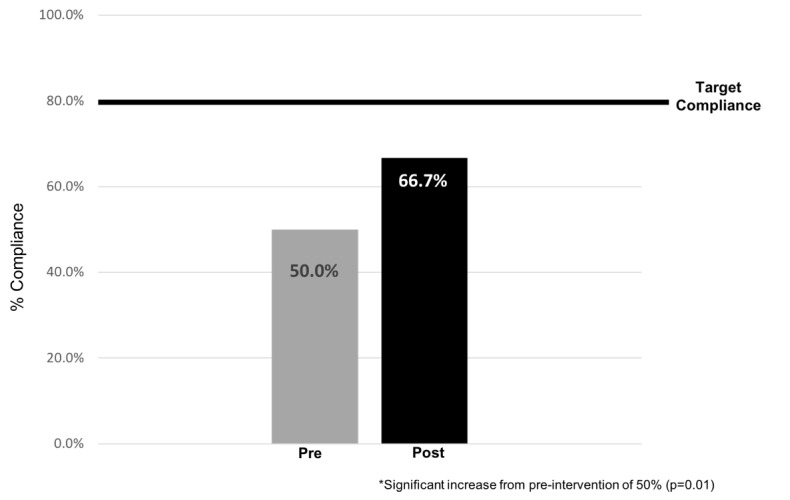
Overall compliance

The overall compliance with guidelines occurred in 36 cases (66.7%), which was a statistically significant increase (p=0.01) from the previously calculated baseline rate of 50%. However, this increase fell short of the authors’ target goal of ≥80% (Figure 1).

**Figure 2.**
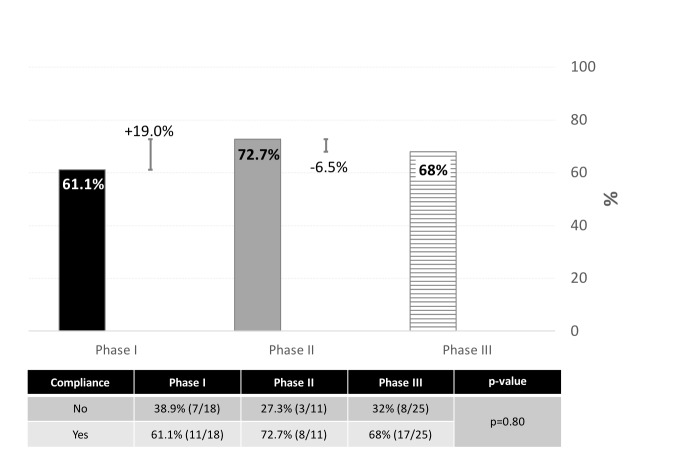
Overall compliance by study phase

There was an initial increase to 61.1% compliance after the first phase of the project, which increased to 72.7% (percent increase of 19%) compliance after Phase 2 (Figure 2). However, compliance dipped slightly to 68% (percent decrease 6.5%) during the third phase of the initiative where no intervention related activity took place. There was no significant difference in compliance rates among the various intervention time points (p=0.80; Figure 2).

### Antibiotic Use

Overall frequency of antibiotic use occurred in 11 cases (20.4%). Of the encounters where guidelines were not adhered to, four cases (22.2%) resulted in an antibiotic prescription. Of the encounters where the guidelines were adhered to, seven cases (19.4%) resulted in an antibiotic prescription. The most common oral antibiotic was cephalexin (72.7%) which was prescribed over a period of 5 days. Other antibiotics included ciprofloxacin (9.1%), amoxicillin-clavulanate (9.1%), and trimethoprim-sulfamethoxazole (9.1%).

## DISCUSSION

The primary goal of this study was to increase compliance to the IDSA guidelines for treating ASB to > 80% post-initiative from a baseline of 50% using a stepwise educational intervention approach. The educational literature had previously established spaced repetition as an effective means to educate, which was the approach used in our intervention.^10,11^ We spaced out each time point with one month of education followed by three months of no intervention. The result was improved compliance reaching 66.7% over the course of the initiative but fell short of our >80% target compliance goal.

The initial educational intervention in Phase I was a classic form of direct education, including a 20-minute teleconference presentation completed by infectious disease experts covering the IDSA guidelines along with case studies, distribution of literature and algorithmic approach, showing an absolute increase from baseline of 11.1% at three months post intervention.

The second intervention with changes involving the EHR, essentially acting as a hard-stop prompt, encouraged clinicians to consider if a urine culture is appropriate which would suggest that the patient had urinary symptoms or qualified for treatment should results return consistent with ASB as defined previously. Changes to EHR ordering systems have been made at other institutions demonstrating similar improvements in antibiotic stewardship.^13,14^ These changes in other settings involved decision support tools and order sets that were implemented, showing promising changes in stewardship efforts. In our study, an absolute increase of 11.6% was seen from Phase I to Phase II, like the increase seen from baseline to post-phase I intervention.

The last phase of the intervention, no education, resulted in a slight decrease of 4.7%. This reiterates the importance of a continuing repetitive educational model with various touch points to readdress various issues in the medical system, notably in this case appropriately addressing ASB.^10,11^ This result may suggest that starting with a passive intervention, requiring participants to review educational materials, followed by an active change requiring provider engagement, may be a more effective strategy.

A prior cohort study by Petty et al. published in 2020 assessed IDSA ASB compliance involving patients admitted through the ED, seeking to evaluate patterns of testing and treatment initiated by emergency medicine providers along with outcomes.^2^ This was a robust study including 43 hospitals identifying 2,461 patients with ASB, 74.4% of which received antibiotics. This study group found that predictors of non-adherent treatment included patient histories of spinal cord injury, dementia, incontinence, altered mental status, presence of a urinary catheter, abnormal urinalysis, and leukocytosis. Patients who received antibiotics in this context overtly had longer periods of hospitalization without improved outcomes and in some cases, development of *C. diff* infection.

Our project certainly did not include nearly as many subjects, but altered mental status was also commonly found in our project to be a typically identified reason for initiating antibiotics in ASB, contributing to non-adherence. Testing and subsequent treatment of ASB often occurred at the level of the ED encounter versus other outpatient settings prior to this visit, which also mirrored findings by Petty et al.^2^

### Project Limitations

Although the initial intervention required participation of ED clinicians to review provided materials, their subsequent participation was not entirely mandatory. Although the second project phase did require clinicians to select an indication for ordering a urine culture before an antibiotic order could be placed, bypassing this step may have been possible by setting up an order preference with a preselected indication in the EHR, avoiding the hard-stop prompt when placing orders. This smaller sample project was certainly limited by the number of validated ASB cases.

## CONCLUSION

Future more rigorous projects to examine factors influencing appropriate treatment of ASB in ED settings are needed. Based on our results, identifying ideal strategies to change treatment patterns of ED clinicians to align with guidelines appears to be key to addressing this issue. A stepwise approach to completing such goals with ongoing spaced repetition educational initiatives may be required achieve and maintain compliance goals.

### Conflict of Interest

None
